# Concomitant Infection with *Leishmania donovani* and *L. major* in Single Ulcers of Cutaneous Leishmaniasis Patients from Sudan

**DOI:** 10.1155/2014/170859

**Published:** 2014-03-12

**Authors:** A. M. Babiker, S. Ravagnan, A. Fusaro, M. M. Hassan, S. M. Bakheit, M. M. Mukhtar, G. Cattoli, G. Capelli

**Affiliations:** ^1^Istituto Zooprofilattico Sperimentale delle Venezie, Viale dell'Università, 10-35020 Legnaro (PD), Italy; ^2^Department of Public Health and Comparative Pathology, University of Padua, Viale dell'Università, 16-35030 Legnaro, Italy; ^3^Tropical Medicine Research Institute, National Centre for Research, P.O. Box 1304, Khartoum, Sudan; ^4^Institute of Endemic Diseases, University of Khartoum, Medical Campus, Qasr Avenue, P.O. Box 45235, 11111 Khartoum, Sudan

## Abstract

In Sudan human leishmaniasis occurs in different clinical forms, that is, visceral (VL), cutaneous (CL), mucocutaneous (ML), and post-kala-azar dermal leishmaniasis (PKDL). Clinical samples from 69 Sudanese patients with different clinical manifestations were subjected to a PCR targeting the cytochrome oxidase II (COII) gene for *Leishmania* species identification. Mixed infections were suspected due to multiple overlapping peaks presented in some sequences of the COII amplicons. Cloning these amplicons and alignment of sequences from randomly selected clones confirmed the presence of two different *Leishmania* species, *L. donovani* and *L. major*, in three out of five CL patients. Findings were further confirmed by cloning the ITS gene. Regarding other samples no significant genetic variations were found in patients with VL (62 patients), PKDL (one patient), or ML (one patient). The sequences clustered in a single homogeneous group within *L. donovani* genetic group, with the exception of one sequence clustering with *L. infantum* genetic group. Findings of this study open discussion on the synergetic/antagonistic interaction between divergent *Leishmania* species both in mammalian and vector hosts, their clinical implications with respect to parasite fitness and response to treatment, and the route of transmission with respect to vector distribution and or adaptation.

## 1. Introduction

Leishmaniasis is a vector-borne parasitic disease caused by intramacrophage protozoa of the genus* Leishmania*, transmitted generally by at least 30 species of sand flies (either* Phlebotomus *or* Lutzomyia* genera) and rarely by congenital and parenteral routes [1, 2]. Depending on the species of* Leishmania* involved, humans and a wide range of mammals can act as reservoirs [3]. At least four major clinical forms of leishmaniasis are recognised: cutaneous leishmaniasis (CL), either diffused or localized, mucocutaneous leishmaniasis (ML), visceral leishmaniasis (VL), fatal if untreated, and the post-kala-azar dermal leishmaniasis (PKDL).

The disease is endemic in the tropical and subtropical regions of 88 countries [4, 5]. Population displacements and increasing cases of* Leishmania*/*HIV* coinfection brought new dramatic concerns to the disease especially in developing countries [6]. Leishmaniasis-affected individuals are estimated to be about 12-13 million worldwide with an annual incidence of 1–1.5 million new cases of CL and 500,000 of VL [4, 7]. However, the real burden of leishmaniasis is greatly underestimated and a substantial number of cases remain unrecorded and misdiagnosed [8, 9]. In Sudan leishmaniasis represents a serious health problem and outbreaks occur periodically causing a high number of victims [10–12]. Many different clinical forms of Leishmaniasis coexist. CL is caused by* L. major* and transmitted by* Phlebotomus papatasi*. It is endemic in the central and northern part of the country [13], However, recently, CL due to* L. donovani* has been well documented [14]. VL is the most serious form caused by* L. donovani*, transmitted by* Phlebotomus orientalis* [15, 16], and endemic in the eastern part of the country [17, 18]. However, scattered cases have been reported from areas not known to be endemic in the southern, northern, and western parts of the country [11]. Both anthroponotic transmission and zoonotic transmission of VL were thought to occur [15, 19–21]. PKDL occurs in high rates during or shortly after treatment. At least 50% of VL patients develop PKDL and this percentage is higher than in any other VL endemic area [22–24]. PKDL patients are thought to act as human reservoir for the parasite [25]. Sudanese mucosal leishmaniasis (SML) is a rare and particular form of ML. Unlike ML, SML starts usually as primary mucosal disease, without being preceded or accompanied by cutaneous lesions. SML occurs in areas endemic for VL [26, 27].* Leishmania*/HIV coinfection is a growing concern particularly in the East [6].

Few studies have been reported on the naturally mixed infection with different species or strains of* Leishmania* in immunocompetent patients. However, natural infection with more than one* Leishmania*, especially where foci of two species overlap, is more prevalent than previously reported. Nonetheless, the degree of laboratory detection of such phenomenon remains unclear [28].

The general aim of this study was to genetically characterize* Leishmania* isolates from patients with various clinical manifestations. Here we report for the first time the detection of three cases of mixed* L. donovani* and* L. major* infections in three out of five patients diagnosed as cutaneous Leishmaniasis patients with no apparent clinical symptoms of VL.

## 2. Materials and Methods

### 2.1. Clinical Samples

One hundred and eight samples composed of blood (HB), bone marrow (HBM), lymph node aspirates (HLN), extracted DNA and cultured parasites were obtained from 69 patients clinically suspected of leishmaniasis. From 21 of these patients, HBM, HB, and HLN aspirates were taken. Seven HB samples and one HLN were collected from eight symptomatic VL patients at Médecins Sans Frontières-Suisse (MSF-CH) Leishmaniasis clinic inside the local Tabarak Allah hospital (Gedarif, Sudan). Other 20 HB and 5 HMB were collected from south Gedarif (Table 1). All described samples were collected from known VL endemic areas. Clinical samples were spotted on Whatman filter paper grade 3; each filter paper sample was stored in a separate polyethylene bag at room temperature till further analysis.

Five samples were taken from five patients presented at the Institute of Endemic Disease, University of Khartoum, Sudan (IEND), with typical CL ulcers. In addition, ten extracted genomic DNA of seven VL, one CL, one ML, and one PKDL samples were kindly provided by the same institute (Table 1). CL patients were not from VL known endemic areas and did not show clinical symptoms of VL. A single cutaneous aspirate from the edges of the ulcer was inoculated into bottles containing biphasic media (NNN) and incubated at 24°C. Cultures were examined microscopically for parasite growth and the successfully cultured isolates were mass-cultured in 200 mL of RPMI-1640 supplemented with 10% foetal calf serum (FCS) and 1% of Penicillin/Streptomycin solution.* L. major* MON 25, kindly provided by the* Leishmania* reference centre, Italy, was included in this study.

## 3. Biomolecular Assay

### 3.1. DNA Extraction

DNA of the cultured parasites was extracted using phenol/chloroform method as described elsewhere [29].

Filter papers with spotted biological material (lymph node or bone marrow aspirates or blood) were punched out with a paper puncher. To prevent DNA contamination among samples, clean sheet of paper was sprayed with DNA decontaminant [30] using MicroSol 3+ solution and was punched several times as recommended by the World Health Organization (available at http://www.who.int/hiv/topics/drugresistance/dbs_protocol.pdf) for HIV patients. DNA extraction was performed using QIAamp DNA Mini Kit according to the manufacturer's instructions.

### 3.2. Real-Time PCR Assay

As an initial screening, a real-time PCR was conducted to investigate the presence of* Leishmania complexes* (*L. viannia, L. mexicana, L. donovani/infantum*, and* L. major*) in all samples. The primers Lid-f and Lid-r which generate a 80 bp fragment of the GPI gene [31] were used with the probe TaqMan MGB Lid-probe 5-ATCGGCAGGTTCT-3 labeled with the fluorescent reporter dye FAM (6-carboxyfluorescein) at the 5′end and with the fluorescent quencher dye TAMRA (tetra-methyl carboxyrhodamine). Real-time PCR was performed in a final volume of 20 *μ*L containing 3 *μ*L of DNA, 10 *μ*L of FastStart TaqMan Probe Master (Rox)1X (Roche Mannheim, Germany), 0.4 *μ*M of both primers, and 0.3 *μ*M of the probe. The thermal cycling profile consisted of an initial activation at 95°C for 10 min, followed by 45 cycles each consisting of denaturation at 95°C for 15 sec and annealing/extension at 60°C for 30 sec. Negative (sterile water) and positive (DNA extracted from* L. infantum* MON-1, MHOM/TN/80/IPT1, IZSVe. Italy) controls were included in each run of real-time PCR reaction. Real-time PCR was carried out on a 7900HT fast real-time PCR system (Applied Biosystems).

To determine the genetic profile of* Leishmania spp*., DNA from each positive sample was subjected to the amplification of the cytochrome oxidase II gene.

### 3.3. Cytochrome Oxidase II PCR

PCR was performed as described previously for the targeted cytochrome oxidase II for all leishmaniasis positive samples [32].

The reaction was performed in a final volume of 50 *μ*L containing 5 *μ*L of DNA, 5 *μ*L of PCR buffer 1X (Applied Biosystems, Foster City, CA), 2 mM of MgCl2 (Applied Biosystems, Foster City, CA), 0.4 *μ*M of each primer, 0.2 mM of dNTPs (Applied Biosystems, Foster City, CA), and 2 U of AmpliTaq Gold DNA polymerase (Applied Biosystems, Foster City, CA). Amplifications were carried out in a GeneAmp PCR System 9700 thermal cycler (Applied Biosystems, Foster City, CA) with the following thermal cycling profile: denaturation for 10 min at 95°C, followed by 35 cycles each consisting of 30 sec at 94°C, 30 sec at 50°C, 45 sec at 72°C, and a final extension step for 7 min at 72°C. Negative and positive controls were included in each run of PCR as described above.

All the PCR products were analysed on 7% acrylamide gel, visualized by silver staining, and subsequently subjected to sequencing. PCR products were sequenced using the Big Dye Terminator v3.1 cycle sequencing kit (Applied Biosystems, Foster City, CA, USA). The products of the sequencing reactions were purified using PERFORMA DTR Ultra 96-Well kit (Edge BioSystems, Gaithersburg, MD, USA) and sequenced in a 16-capillary ABI PRISM 3130xl Genetic Analyser (Applied Biosystem, Foster City, CA, USA). Sequence data were assembled and edited with SeqScape software v2.5 (Applied Biosystems, Foster City, CA, USA). Sequences obtained were aligned and studied. The sequences which showed overlapping nucleotides peaks were subjected to cloning.

### 3.4. Internal Transcribed Spacer (ITS) PCR

ITS PCR was performed in the three cutaneous samples that showed overlapping nucleotides peaks in the COII sequences. The ITS region (1044 pb nucleotides) was amplified with the* Leishmania* specific primers: LITSR [33] and a new designed primer ITSRR (5′-AGAGTGAGGGCGCGGATA-3′) that amplify* L. donovani complex* and* L. major*.

The reaction was performed in a final volume of 50 *μ*L containing 0.5 *μ*L of DNA, 5 *μ*L of PCR buffer 1X (Applied Biosystems, Foster City, CA), 2 mM of MgCl2 (Applied Biosystems, Foster City, CA), 0.4 *μ*M of each primer, 0.2 mM of dNTPs (Applied Biosystems, Foster City, CA), and 2.5 U of AmpliTaq Gold DNA polymerase (Applied Biosystems, Foster City, CA). Amplifications were carried out in a GeneAmp PCR System 9700 thermal cycler (Applied Biosystems, Foster City, CA) with the following thermal cycling profile: denaturation for 10 min at 95°C, followed by 35 cycles each consisting of 30 sec at 94°C, 30 sec at 50°C, 1 minute at 72°C, and a final extension step for 10 min at 72°C. Negative and positive controls were included in each run of PCR as described above.

All the PCR products were analyzed on 7% acrylamide gel and visualized as described above and cloned.

### 3.5. Cloning Assay

The three PCR products of the COII and the three PCR products of the ITS genes were cloned separately into the PCR-II vector using a dual-promoter TOPO TA cloning kit (Invitrogen, Carlsbad, CA) according to the manufacturer's instructions. Plasmids with the desired inserts were isolated from positive* Escherichia coli* colonies by using a GenElute plasmid miniprep kit (Sigma-Aldrich, St. Louis, MO). DNA extracts from at least 30 randomly selected colonies were sequenced as described above.

### 3.6. Phylogenetic Analysis

Sequencing data were assembled and edited with SeqScape software v2.5 (Applied Biosystems) and analyzed. Phylogenetic analysis was conducted by using the neighbor-joining method with 1000 bootstrap replicates implemented in MEGA 5 software [34].

## 4. Results

Overall, the diagnosis of leishmaniasis was confirmed by real-time PCR in 40 patients out of 69 (58%). The real-time PCR confirmed 58 samples out of 110 (53%) as* Leishmania* positive. The positivity in real-time PCR was higher for HLN samples (66%) compared to HBM (57%) and HB (29%).

### 4.1. Cytochrome Oxidase II Sequence Analysis and Phylogeny

#### 4.1.1. Direct Sequence Analysis

**Figure 1:**
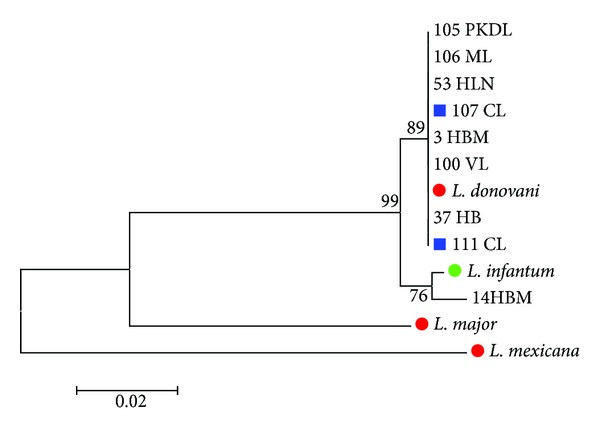
*Phylogenetic tree of COII sequences. *Phylogenetic tree based on a fragment of 570 bp of COII gene of* Leishmania* parasite. Sequence dataset was analysed using MEGA 5, the neighbor-joining (NJ) method, and bootstrap analysis (1,000 replicates) based on the ClustalW algorithm. Significant bootstrapping values (>70%) are shown on the nodes. Only representative sequences were shown. Number indicates the sample number, HBM indicates human blood marrow origin sequence, HLN indicates lymph node origin sequence, and HB indicates human blood origin sequence used in this study. Blue square: sequence numbers 107 and 111 originated from skin culture amplificates and associated with CL patients; red circle:* Leishmania* GenBank reference sequence (*L. donovani* GenBank accession number AY660023,* L. major* GenBank accession number AF63316, and* L. mexicana GenBank accession number HQ586845.1*); green circle: our positive control* L. infantum* MHOM/TN/80/IPT1 used in this study.

Samples associated with VL, PKDL, and ML clinical forms showed a unique sequence pattern with 100% genetic homology to* L. donovani* AY660023.1 reference sequences deposited in GenBank (Figure 1). One sequence associated with VL clinical symptoms (14 HBM) showed the highest identity (98.9%) to* L. infantum* MHOM/TN/80/IPT1 reference sequence and a similarity of 98.1% to the abovementioned* L. donovani *GenBank reference sequence (Figure 1). The genetic homology between* L. donovani* GenBank reference sequence and* L. infantum* sequence used in this study was 98.7%.

Two sequences associated with CL samples clustered within the* L. donovani* group (numbers 107 and 111) with 100% genetic identity (Figure 1).

**Figure 2:**
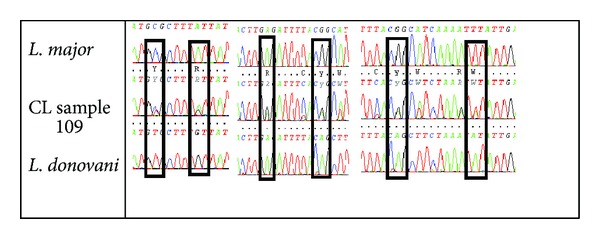
Schematic demonstration of COII sequences obtained by direct sequencing of one CL isolate (sample number 109) showing overlapping of nucleotides in positions 93, 99, 153, 159, 162, and 173 (position according* to L. donovani* GenBank accession number AY660023); CL sequence compared* to L. major* MON 25 and to our 3HBM sequence that gave 100% identity to* L. donovani* (GenBank accession number AY660023).

The remaining three CL sequences showed overlapping nucleotides peaks (Figure 2) and therefore were cloned.

#### 4.1.2. Cloning Sequences Analysis

Thirty-four, twenty-nine, and thirty-six sequences were obtained from cloning of CL samples numbers 108, 109, and 110, respectively.

**Figure 3:**
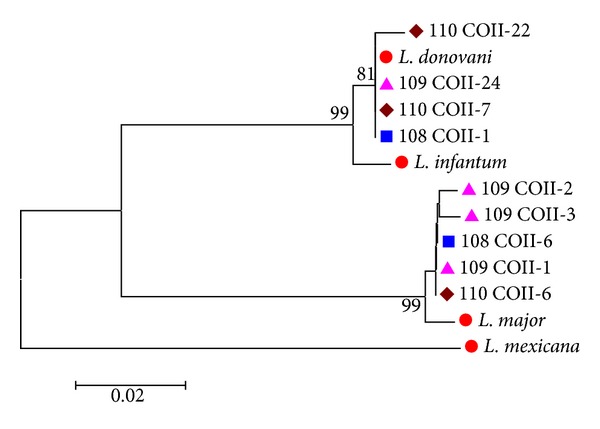
Phylogenetic tree of COII cloning sequences. Phylogenetic tree based on a fragment of 570 bp of COII gene of* Leishmania* parasite. Sequence dataset was analyzed using MEGA 5, the neighbor-joining (NJ) method, and bootstrap analysis (1,000 replicates) based on the ClustalW algorithm. Significant bootstrapping values (>70%) are shown on the nodes. Blue square: colonies numbers 1 and 6 as representative sequences of 108 cytochrome oxidase II cloned sample; purple triangle: colonies numbers 1, 2, and 3 as representative sequences of 109 cytochrome oxidase II cloned sample; dark red lozenge: colonies numbers 6, 7, and 22 as representative sequences of 110 cytochrome oxidase II cloned sample; red circle:* Leishmania* GenBank reference sequence (*L. donovani* GenBank accession number AY660023;* L. major* GenBank accession number EF633106;* L.mexicana* GenBank accession number HQ586845.1 and our positive control* L.infantum* MHOM/TN/80/IPT1).

Phylogenetic analysis of the abovementioned colonies (Figure 3) revealed two different sequence patterns, corresponding to* L. donovani* with genetic similarity range between 99.5% and 100% to* L. donovani AY660023 *GenBank reference sequence and to* L. major* with genetic similarity ranging between 98.9 and 99.3% to* L. major* EF633106 GenBank reference sequence. The genetic similarity between the two GenBank reference sequences used here was 89.4%.

#### 4.1.3. ITS Sequence Analysis and Phylogeny

**Figure 4:**
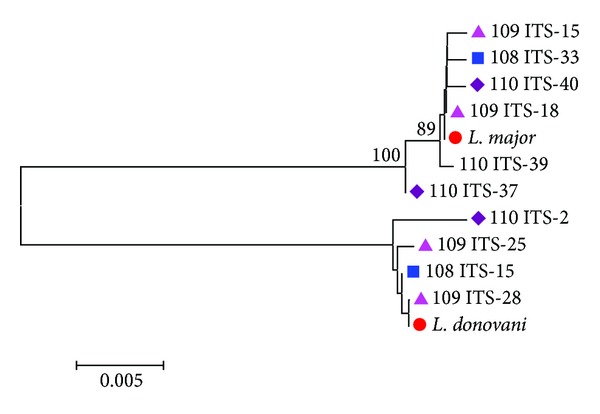
Phylogenetic tree of ITS cloning sequences. Phylogenetic tree (unrouted) based on a fragment of 970 bp of ITS gene of* Leishmania* parasite. Sequence dataset was analyzed using MEGA 5, the neighbor-joining (NJ) method, and bootstrap analysis (1,000 replicates) based on the ClustalW algorithm. Significant bootstrapping values (>70%) are shown on the nodes. Blue square: colonies numbers 15 and 33 as representative sequences of 108 ITS cloned sample; pink triangle: colonies numbers 15, 18, 25, and 28 as representative sequences of 109 ITS cloned sample; purple lozenge: colonies numbers 2, 37, 39, and 40 as representative sequences of 110 ITS cloned sample; red circle:* Leishmania* GenBank reference sequence (*L. donovani* GenBank accession number AJ634357;* L. major* GenBank accession number FJ75339).

Twenty-three, fourteen, and eighteen sequences were obtained from cloning of samples numbers 108, 109, and 110, respectively. As for COII, the phylogenetic analysis of the ITS cloning sequences revealed two different sequence patterns, corresponding to* L. donovani *with 99.5–100% genetic similarity to* L. donovani* AJ634357 GenBank reference sequence and 99.8–100% to* L. major* FJ753391 GenBank reference sequence. The genetic similarity between* L. major* and* L. donovani* used in this study was 93.5% (Figure 4).

All the representative sequences showed in the trees (Figures 1, 3, and 4) have been submitted to GenBank (accession numbers from KF815198 to KF815226).

## 5. Discussion

The COII and the ITS genes gave concordant results and confirmed naturally mixed infection with* L. major* and* L. donovani *from a single cutaneous aspirate in the three CL patients who presented no evident VL clinical signs.

*L. major* and* L. donovani* have different transmission vectors and distinct biologic properties and epidemiologic features and both have been reported as etiologic agents of CL in Sudan.

CL due to* L. major* LON1 is transmitted by* P. papatasi* in arid and semi-arid areas and circulates among rodents [35], while the vector that transmits CL due to* L. donovani* has not been established yet.

Coinfection with different* Leishmania* has been reported in closely related* Leishmania* species such as* L. braziliensis, L. panamensis *[36],* L. major,* and* L. arabica* [37]. The only two reports on concomitant natural infection with* L. donovani* and* L. major *were reported in Iraq where the two species were isolated from two different sites, the bone marrow and cutaneous lesions from kala-azar suffering patients [38] and in Kenya where isolation was achieved in a spleen aspirate culture from a clinically relapsed kala-azar suffering patient [39]. Concomitant infection with divergent* Leishmania* species has also been reported. The two species* L. infantum* and* L. major* have been isolated from the spleen of VL/HIV immune-compromised patient [40];* L. chagasi* and* L. amazonensis* have been isolated from a diffuse cutaneous leishmaniasis case in Bolivia [41].

In our study, the three coinfected patients were presented with typical localized cutaneous ulcers, with neither evident signs of systemic illness nor immunosuppression or VL. All three patients originated from a VL non endemic area and had no history of travelling to a known VL endemic area. However, even if visceral involvement or immunosuppression was not clinically suspected, no other clinical or laboratory examinations were carried out.

The occurrence and frequency of natural infection with more than one* Leishmania*, especially where foci of two species overlap, are believed to be more prevalent than reported. It has been demonstrated that in co-cultivation of more than one* Leishmania* the dominant species tend to inhibit the growth of the other; consequently the degree of laboratory detection of such phenomenon remains unclear and likely underestimated [28, 42].

In our case, the two species were likely maintained because the cultured parasites were harvested and extracted during early growth at the third day of culture. However, after cloning, the number of colonies related to* L. major* genetic group was higher compared to that related to* L. donovani* genetic group. This may suggest the dominance of* L*.* major* species in the co-culture; however, it is also possible that the PCR conditions were more efficient towards* L. major* genome.

Assumption of circulation of more than one strain/hybrid of* L. major* and* L. donovani* was demonstrated by the presence of more than one sequence pattern of* L. major* and* L. donovani* within the same patient (Figures 3 and 4). Recent studies have demonstrated the presence of genetically different populations of* P. papatasi* in Sudan [43] that could be able to transmit different strain/hybrids. Moreover, high polymorphism has been attributed to* L. major* causing cutaneous diseases in Iran [44]. The hypothesis of the circulation of more than one strain/hybrid of* L. donovani* among Sudanese patients is supported by our findings after analyzing clones of the ITS gene and the GP63 gene from different VL patients (data not shown). However, the intraspecific variations of* Leishmania* species among Sudanese patients and its correlation with clinical signs need to be explored.

The other two cutaneous cases were identified to be caused by* L. donovani* only as reported by other the authors [14].

The transmission route of naturally mixed infection remains debated.* P. papatasi* is not a vector of visceral Leishmaniasis and is refractory to the infection by* L. infantum* and* L. donovani* [45, 46]. However, an experimental evidence of* P. papatasi* infection with hybrid strains of* L. infantum/L. major* causing visceral disease in HIV-positive patients has been documented [47]. Based on these results,* P. papatasi* has been suggested as vector of this hybrid in nature [47].

All the other cases of VL (except one case), ML, and PKDL were caused by* L. donovani*; however, the limited numbers of ML and PKDL samples did not allow investigating whether other species of* Leishmania* were involved.

Cytochrome oxidase II gene confirmed its good capability to discriminate among different species of* Leishmania*. Previously, an extensive research on VL based on the isoenzyme classification led to the conclusion that* L. donovani* is the only cause of visceral leishmaniasis in Sudan [48]. In our study, cytochrome oxidase II revealed one sample with sequences very similar to* L. infantum* genetic group and further studies are needed to confirm this finding. However, the correspondence between genetic and isoenzyme classifications is still debated and a revision of the taxonomy of the genus* Leishmania* has been proposed by other researchers [49, 50].

## 6. Conclusion

To the best of our knowledge this is the first report of* L. donovani* and* L. major* co-infection in Sudan. Additionally no other cases of such co-infection from the same cutaneous sample were reported.

This finding has important implications regarding the diagnosis, the choice of the most appropriate therapy, and the possibility of developing drug resistance.

The limited number of cutaneous samples examined does not allow predicting the frequency of this coinfection. However, the presence of* L. donovani* in all the five cutaneous samples suggests caution in the followup of CL patients who could later develop VL symptoms. Many aspects of leishmaniasis in Sudan still need to be explored, such as the prevalence of different species and vectors and the competence of* Phlebotomus* spp. in transmitting different* Leishmania* species.

## Figures and Tables

**Table 1:** Provenance, numbers, and types of samples collected included in the study.

Geographic origin	Sample type	Number of samples	Leishmaniasis diagnosis	Clinical manifestation
Central Gedarif city	HBM*	21	Symptomatic	VL
	HB*	21	Symptomatic	VL
	HLN*	18	Symptomatic	VL
Tabaraka Allah H	HB	7	Serological	VL
	HLN	1	Serological	VL
South Gedarif city	HBM	5	Symptomatic	VL
	HB	20	Symptomatic	VL
Khartoum-IEND	DNA	7	Culture	VL
	DNA	1	Culture	PKDL
	DNA	1	Culture	ML
	DNA	6	Culture	CL

HBM: human bone marrow; HB: human blood; HLN: human lymph node; IEND: Institute of Endemic Diseases.

*Samples from the same patients.
